# Regulation of Immune Cell Migration by Sphingosine-1-Phosphate

**Published:** 2015-12-31

**Authors:** A. Kumar, JD. Saba

**Affiliations:** 1 Department of Biochemistry, All India Institute of Medical Sciences [AIIMS], Saket Nagar, Bhopal 462 020, India; 2 Children’s Hospital Oakland Research Institute, 5700 Martin Luther King Jr. Way, Oakland, California 94609, USA

**Keywords:** Sphingosine-1-Phosphate, Sphingosine Phosphate Lyase, Sphingolipid, Lymphocyte Trafficking, Thymic Egress, S1PR1, Lymphatic

## Abstract

Sphingosine-1-phosphate [S1P] is a potent bioactive sphingolipid molecule. In response to a stimulus, S1P is produced intracellularly by the action of two sphingosine kinases, and then it is exported to the extracellular environment or acts as an intracellular second messenger. S1P binds to its cognate G-protein coupled receptors, which are known as S1P receptors. There are five S1P receptors that have been identified in vertebrates. By activating S1P receptors, S1P controls a variety of physiological and pathological processes including cell migration, angiogenesis, vascular maturation, inflammation, and invasion, metastasis, and chemoresistance in cancer. S1P has emerged as a critical regulator of leukocyte migration and plays a central role in lymphocyte egress from the thymus and secondary lymphoid organs. In the current review article, we summarize the current understanding of the emigration of lymphocytes and other leukocytes from bone marrow, thymus and secondary lymphoid organs to the circulation, as well as the clinical implications of modulating the activity of the major S1P receptor, S1PR1.

## Introduction

Sphingosine-1-phosphate [S1P] is a sphingolipid metabolite and a potent signaling molecule that regulates diverse cellular processes including cell proliferation, survival, differentiation and migration [1–3]. Intense research by many groups has provided a comprehensive understanding of the role of S1P signaling in diverse physiological processes. These include but are not limited to metazoan and mammalian development, reproduction, angiogenesis, vascular maturation, inflammation, the response to ischemic injury, leukocyte migration, and cancer progression, metastasis, invasiveness and chemoresistance [3]. S1P exerts most of its biological actions by serving as a ligand for five G protein-coupled receptors [GPCRs] known as S1P receptors 1-5 [S1PR1-5]. After the discovery that the phosphorylated form of FTY720, an immunosuppressive compound, is an analog of S1P and binds to four of the five S1PRs, the major role that S1P signaling plays in lymphocyte trafficking was recognized and the potential to target this pathway for therapeutic benefit envisaged [4–6]. In fact, FTY720 [Gilenya] has now been approved by the Food and Drug Administration for the treatment of multiple sclerosis [4] and is being evaluated in the treatment of other autoimmune diseases, demonstrating the clinical utility of targeting S1P signaling for the purpose of immunomodulation [4,7]. Although the regulation of T cell egress from the thymus by S1P/S1PR1 signaling is the most well-characterized and pharmacologically exploited of its known functions, the regulation of hematopoietic cell trafficking is a leitmotif of S1P signaling. In addition to T cell egress and trafficking, S1P also regulates B cell, natural killer [NK] cell and hematopoietic stem cell [HSC] trafficking, the development and differentiation of leukocytes, mast cell degranulation, antigen presenting cell functions and other immune responses [8]. Some of the important functions of S1P in the immunity are summarized in Table 1.

In this review, we summarize the roles of S1P signaling in regulating immune cell trafficking and discuss recent advances that have shed light on the underlying mechanism by which S1P controls these processes.

## Synthesis and Metabolism of S1P

**Figure 1:**
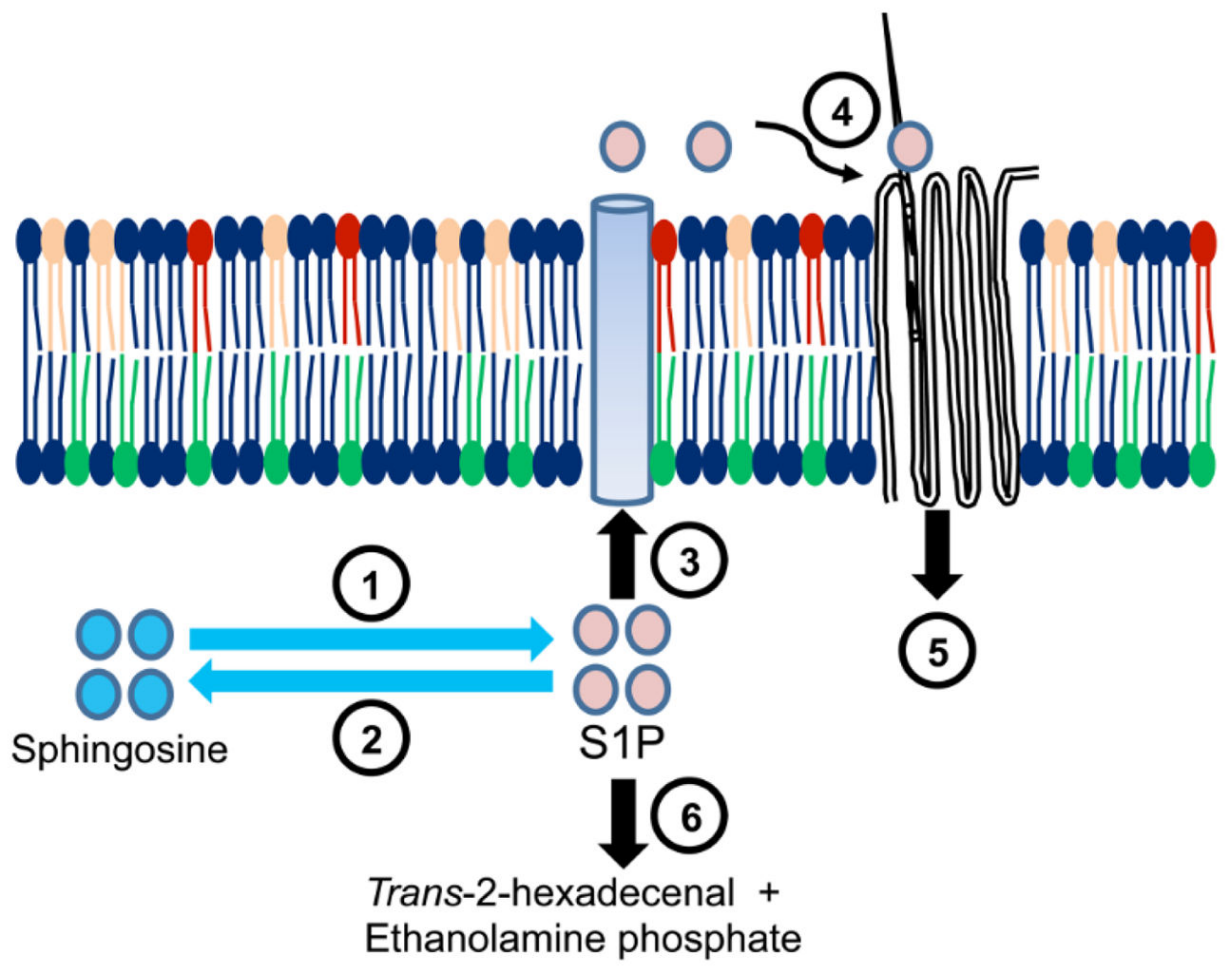
S1P metabolism, export and mechanism of action. (1) SphK1 and SphK2 phosphorylate sphingosine to generate S1P, which can be dephosphorylated (2) by the actions of LPP1-3 or SPP1-2. S1P can be exported through various transporter proteins (3) including Spns2 and ABC transporters. Once released from the cell, S1P can activate (4) S1PRs present on the same cell or neighboring cells, leading to initiation of downstream signaling (5). High S1P lyase (6) activity in the thymus and secondary lymphoid tissues keeps tissue levels of S1P low.

Sphingosine kinase 1 [SphK1] and sphingosine kinase 2 [SphK2] catalyze the phosphorylation of sphingosine to form S1P (Figure 1). Both enzymes phosphorylate sphingosine, whereas SphK2 exhibits broader substrate specificity and can also phosphorylate phytosphingosine and FTY720 [9,10]. Sphingosine is produced mainly by two pathways: de novo sphingolipid biosynthesis or by breakdown of ceramide. In de novo biosynthesis, the first step is the condensation of L-serine and palmitoyl-CoA through the action of serine palmitoyltransferase to form 3-ketodihydrosphingosine, which is then reduced to dihydrosphingosine, followed by acylation to form dihydroceramide. After desaturation of dihydroceramide to produce ceramide, sphingosine can be liberated by ceramidase action [11,12]. In the salvage pathway, ceramide is produced after the hydrolysis of sphingomyelin, a membrane sphingolipid [12].

S1P levels in the tissues and plasma are tightly regulated. SphK1 is the primary enzyme responsible for S1P generation and is mainly localized in the cytosol. After activation, SphK1 translocates to the plasma membrane. SphK1-generated S1P has been implicated in many pathological conditions including vascular permeability, angiogenesis and inflammation [13–16]. Compared to copious experimental evidence characterizing SphK1 and implicating it in physiology and disease, much less is known about the regulation and function of SphK2. SphK2 is localized in mitochondria, the nucleus and the endoplasmic reticulum and regulates apoptosis and gene expression [9,10,17,18]. There are six enzymes that are recognized to catabolize S1P in tissues and the circulation (Figure 1). Two S1P-specific phosphates, S1P phosphatase 1 and 2, are located in the endoplasmic reticulum. These phosphatases dephosphorylate S1P, regenerating sphingosine [19–23]. In addition, three plasma membrane-bound phosphatases known as lipid phosphate phosphatases 1, 2 and 3 [LPP1-3] can act on a broad range of lipid phosphate substrates including ceramide-1-phosphate, S1P, lysophosphatidic acid and phosphatidic acid [24]. S1P lyase is located in the endoplasmic reticulum and degrades S1P irreversibly to ethanolamine phosphate and a long-chain aldehyde [trans-2-hexadecenal], products that can be utilized for phospholipid synthesis [25–27].

## Source and Transport of S1P

S1P is generated in almost all mammalian cell types. In most tissues including thymus and secondary lymphoid organs, S1P levels are low, which is most likely attributed to high levels of S1P lyase activity in these tissues [28–31]. In lymph and blood, S1P concentrations are relatively high [100 nM range in lymph and 1.0 μM range in the blood] in comparison to the tissues. S1P in lymph is produced by radiation-resistant cells of non-hematopoietic origin, presumably lymphatic endothelial cells [32]. In blood, most of the S1P is produced by erythrocytes and platelets [32–34]. In blood, S1P is transported as albumin- and high-density lipoprotein [HDL]-bound forms. To activate its cognate GPCRs in autocrine or paracrine manner, S1P must be exported out of the cell (Figure 1). It is an amphipathic lipid that requires facilitated export from cells. Members of the ATP-binding cassette [ABC] family of transporters, including ABCA1, ABCC1 and ABCG2, have been shown to be involved in the export of S1P from various cell types [35,36]. There is evidence for other members of the ABC transporter family for RBCs and platelets [37–39]. Recently, a novel S1P transporter Spns2 was discovered in zebrafish. Spns2 has been shown to control S1P levels in plasma and lymph by regulating S1P release from endothelial and lymphendothelial cells [39–41].

## Mechanism of Action

Most of the functions of S1P have been attributed to the activation of its cell surface GPCRs [42–44]. To date, five S1PRs EDG1/S1PR1, EDG5/S1PR2, EDG3/S1PR3, EDG6/S1PR4 and EDG8/S1PR5 have been identified in vertebrates [45]. Each S1PR couples to different heterotrimeric G-proteins and activates or inhibits downstream signaling pathways. For example, S1PR1 and S1PR4 couple mainly to Gi, whereas S1PR2 and S1PR3 activate Gi, Gq and G12/13; and S1PR5 binds to Gi and G12/13 [46,47]. Downstream signaling pathways which are activated or inhibited by S1PRs include extracellular signal-regulated kinase [ERK], c-Jun N-terminal Kinase [JNK], phosphatidylinositol 3-kinase [PI3K], phospholipase C, Rac, Rho, cyclic AMP and phospholipase D [3]. In addition to the predominant extrinsic pathway of S1P signaling mediated through cell surface receptors, there is evidence to suggest that S1P may regulate some biological processes including cell survival, DNA damage repair, calcium mobilization, stress response, gene regulation and chemokine production by acting as an intracellular second messenger [48–51]. Only a few intracellular targets of S1P have been identified. These include histone deacetylase [HDAC] 1 and 2 [52], TNF receptor-associated factor 2 [TRAF2] [3], p21-activated protein kinase 1 [53,54] and prohibitin 2 [55].

## S1P in Immune Cell Trafficking

S1P’s role in cell migration has been appreciated since the 1990’s [56]. In the last decade, S1P has emerged as a central mediator in the trafficking of immune cells, including B and T lymphocytes, NK cells, neutrophils, dendritic cells [DCs], macrophages, haematopoietic progenitors and mast cells [57–64]. S1P mediates its effects on cell migration through S1PR activation. There is diversity in the expression pattern of S1PRs in the immune system. [65] S1PR1 is expressed by most immune cells, whereas the other receptors — S1PR2, S1PR3, S1PR4 and S1PR5 — show restricted expression [66] patterns limited to subsets of immune cells [8,48]. For example, T cells express S1PR1 and S1PR4, whereas mast cells and macrophages express S1PR1 and S1PR2. S1PR5 is expressed by DCs and NK cells [65–67]. A tightly regulated spatial and temporal expression of S1PRs and an S1P gradient between the circulation and tissues are crucial for the trafficking of immune cells. Pharmacological, analytical and genetic approaches combined with sophisticated in vivo imaging of immune cells have provided novel insights regarding the role of S1P-S1PR signaling in immune cell trafficking.

### Regulation of thymic settling and thymocyte egress by S1P

Thymic progenitor cells [TPCs] are recruited to thymus from the blood to sustain T cell production. Homing of TPCs in thymus referred to as “thymic settling” occurs in waves over several weeks and is thought to be a gated process. Adhesion molecule P-selectin and chemokine ligand 25 [CCL25] are periodically expressed in the thymic vascular endothelium and are essential for the thymic gate-keeping mechanism [68]. Further, the numbers of peripheral blood lymphocytes directly affect thymic P-selectin expression and TCP, receptivity. S1P acts as an important feedback signal to sense lymphopenia and induce thymic receptivity for TCPs [68,69].

**Figure 2:**
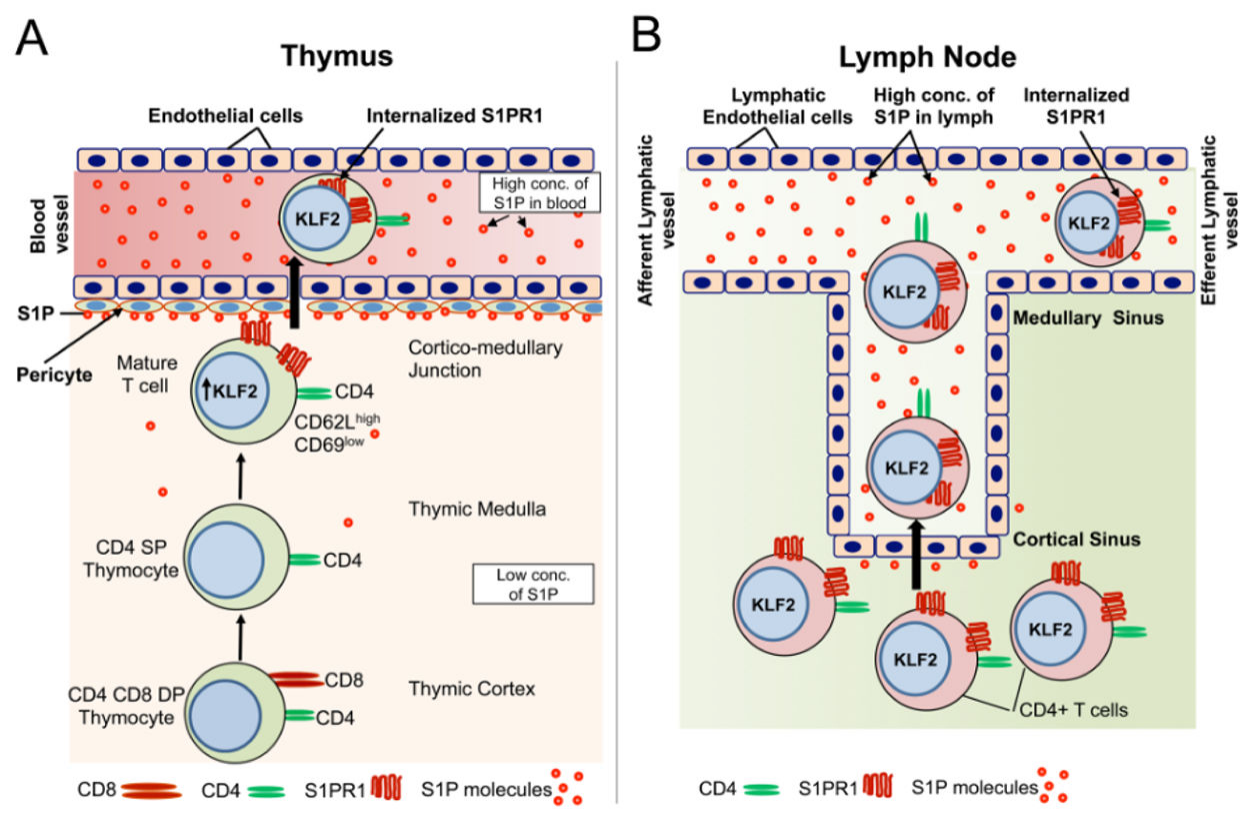
Egress of mature T cells from thymus and peripheral LNs. (A) Thymic T cell egress. CD4+ CD8+ DP thymocytes migrate from the thymic cortex to the medulla, where they differentiate into CD4+ or CD8+ SP thymocytes. Upon maturation, SP thymocytes express high levels of the transcription factor KLF2 in the nucleus (shown in blue), resulting in expression of S1PR1 on their surface. The mature T cells exhibit an S1PR1-positive, CD62 ligand-positive, CD69-low cell surface phenotype. S1P produced by perivascular cells present in the cortico-medullary region activates S1PR1 on mature T cells, leading to their entry into blood. After entering the bloodstream, S1PR1 is internalized into endosomes in the cytoplasm (shown in light green). (B) T cell migration through the LN. T cells randomly migrate and come into contact with the cortical sinus. While probing for the sinus, S1PR1 present on T lymphocytes becomes activated. T cells then detach from the sinus and become caught in lymph flow, which carries them to the medullary sinus and efferent lymphatic.

T cell egress from the thymus is strongly dependent on S1P-S1PR1 signaling (Figure 2). Early TPCs enter into thymus at the cortico-medullary junction and then differentiate into T cell receptor [TCR]-expressing CD4+CD8+ double positive [DP] thymocytes in the cortex (Figure 2A). DP thymocytes undergo positive selection in the thymic cortex and differentiate into CCR7-expressing semi-mature CD4 or CD8 single positive [SP] thymocytes [70,71]. After surviving negative selection, semi-mature SP cells upregulate the expression of the transcription factor Krüppel-like factor 2 [KLF2], which itself has been shown to be under the transcriptional regulation of many factors including Foxo1 and PI3K/AKT. Once expressed in SP cells, KLF2 induces its target gene, S1pr1 [72,73]. Activation of S1PR1 by S1P [48] enables the mature T cells to exit the thymus and enter the circulation [28]. Mature T cells exit the thymus at the corticomedullary junction via blood vessels rather than the lymphatic vessels (Figure 2) [28].

#### Regulation of S1PR1

S1PR1 mRNA expression on T cells is developmentally regulated, increasing by 50-fold between DP and immature SP stages and a further 30-fold between the immature and mature SP stages [66]. In two separate studies, characterization of mice lacking expression of S1PR1 in T cells and hematopoietic reconstitution of wild type mice with KLF2 knockout bone marrow [BM] revealed that both S1PR1 and KLF2 are required for T cell egress, as both mouse models exhibited marked accumulation of mature SP cells in the thymus and a concomitant depletion of mature T cells in the circulation [66,74,75]. Conversely, in a gain-of-function approach, either premature expression of S1PR1 in immature thymocytes or expression in thymocytes lacking KLF2 was sufficient to induce their exit from thymus. is suggests that S1PR1 is the sole KFL2-regulated gene required for T cell egress by overriding the retention signal on thymocytes [62].

Both the expression of S1PR1 on thymocytes and a local S1P gradient [76] are essential for their efficient egress from thymus. In the thymus, levels of S1P of approximately 0.5 pmol/mg wet weight are available to S1PRs.Thymic S1P is maintained at this level by six S1P-catabolizing enzymes [77]. Pharmacological inhibition or genetic disruption of S1P lyase causes ~1000-elevation in thymic [78] S1P levels and disturbs the S1P gradient between parenchyma and the exit site [30,79]. Similarly, genetic disruption of LPP3 also leads to blockage of thymic egress [80]. S1PR1 surface expression is exquisitely sensitive to ligand-mediated internalization, being downregulated by 0.1 to 1 nM S1P [30]. In an environment with high S1P levels such as plasma and lymph, S1PR1 becomes internalized and is not detectable on the cell surface [31,30]. Interestingly, FT720, a synthetic analog of S1PRs is highly active at inducing the internalization, ubiquitylation and subsequent degradation of S1PR1 [81–83].

Surface expression of S1PR1 is also [84] regulated by CD69, a lymphocyte activation marker. CD69 directly interacts with the S1PR1 protein, and the complex of CD69 and S1PR1 is retained inside the cell; if one molecule is present in larger amounts than the other, the excess will appear on the cell surface [61,85]. Mature SP cells normally express intermediate levels of CD69, but S1P signaling causes rapid down-modulation of surface CD69 [86]. Surface expression of CD69 is down-modulated in mature but not in immature SP T cells. In fact, down-modulation of CD69 serves as a sensitive marker of hyperactivation of S1PR1 due to increase S1P levels in thymic parenchyma [30,80]. For example, LPP3 deficiency in thymus did not result in a detectable increase in thymic S1P levels; however, down-modulation of S1PR1 and CD69 was noted on mature T cells and correlated with the blockage of thymic egress observed in this model [80].

It is still not clear how the local gradient of S1P is maintained and regulated, and the source of S1P that is catabolized by S1P lyase or LPP3 is not known. Earlier studies have established that S1P produced by a radiation-resistant source is required for thymic egress [31]. Interesting, an intravital micro-imaging study revealed that mature T cells egress from the thymus via blood vessels but not lymphatic vessels at the corticomedullary junction [62]. Further, it was shown that S1P produced by neural crest-derived pericytes but not blood S1P is required and sufficient for thymic egress [62], suggesting that the pool of S1P required for T cell exit from thymus is compartmentalized and remains protected from S1P-degrading enzymes. Pericytes are stromal cells of the thymus that closely ensheathe blood vessels, but not lymphatics. Mature thymocytes exiting from the thymus are likely to encounter S1P produced by pericytes before engaging the endothelial cells [62]. However, whether S1P produced by vascular endothelium is also required for thymic egress is not known.

Spatial distribution of S1P within the thymus is not known. It has been proposed that extracellular S1P is lower than that in the circulation [28,61], but whether local variations exist in the thymic extracellular S1P levels remains unknown. Whether perivascular S1P produced by pericytes guides T cells towards a portal of egress or whether T cells encounter perivascular S1P in a random hunt for exit points is not known [80]. How the perivascular S1P produced by pericytes remains protected from degradation is not understood. At least five out of six S1P degrading enzymes are expressed in the thymus [27,29,48,80]. LPP3 expression in thymic epithelial cells and endothelial cells but not in hematopoietic cells is required to maintain the S1P gradient and thymic egress [80]. S1P lyase is highly expressed in the thymus, and partial or complete loss of S1P lyase hampers T cell egress, possibly by destroying a local gradient [27,29,79]. The cellular compartment containing the SPL activity required to maintain low thymic S1P remain unanswered. S1P is produced intracellularly and, therefore, must be exported to activate S1PR1. Spinster 2 [Spns2] has emerged as one of main the cell surface transporters facilitating S1P export. Spns2 is required for S1P transport from vascular endothelial cells and promotes thymic egress [87–89]. Two recent studies investigating whether Spns2 regulates plasma or lymph S1P levels have revealed conflicting results [39,40]. Both the studies have shown that loss of Spns2 causes reduced plasma S1P levels. However, the S1P levels in lymph were reduced [39] in one case and in the other were elevated [40]. is discrepancy could be due to different mouse models used in the studies. Mendoza and colleagues have used a conditional knockout mouse model, where Spns2 disruption is driven by Tie2-Cre promoter, whereas Nagahashi et al. [40] have used Spns2 null mice.

## T cell egress from lymphoid and nonlymphoid tissues

After thymic egress into the blood, mature naïve T cells must travel to the secondary lymphoid organs including spleen, lymph nodes [LNs] and Peyer’s patches [PPs]. The egress of T lymphocytes from LNs is well defined [reviewed in ref. 28]. Lymph enters the LNs via afferent lymphatic vessels that are connected to macrophage-enriched medullary sinuses through the subcapsular sinuses (Figure 2B). Lymph percolates through the medullary sinuses before leaving the LN via the efferent lymphatic duct. Lymphocytes exit via medullary sinuses and the efferent lymphatic duct. A multistep model of LN egress has been proposed [90,91], in which cortical sinus ‘probing’ by T cells is followed by S1PR1-dependent entry of lymphocytes into the sinus. Cortical sinuses are present in the LN cortex closer to the surrounding T zone stroma, and they are often blind-ended and initiate near high endothelial venules [HEVs]. The cells then flow into medullary sinuses and the efferent lymph, which contains high levels of S1P produced by lymphatic endothelium [48,61]. After surveying a secondary lymphoid organ for several hours, T cells must exit to travel to other lymphoid organs and continue the surveillance program. Egress from the spleen results in T cell entry into the blood, whereas T cells egress from LNs and PPs directly into the lymph. Lymph carries cells back to the blood via the thoracic and right lymphatic ducts [28]. Egress must be regulated during the immune response. Within the first few hours after an inflammatory stimulus, lymphocyte egress from the responding lymphoid organ is transiently arrested to facilitate antigen encounter by rare cognate cells [28]. The number of antigen-specific lymphocytes must be expanded before they exit as effector cells and traffic to the site of stimuli.

LNs are the most accessible lymphoid organs for intravital microscopy, and not surprisingly T cell egress from LNs has been well studied. Similar to thymic egress, T cell exit from LNs requires not only S1PR1 expression on mature T cells but also the presence of an S1P gradient that is low in LN parenchyma and high at the exit site [28]. Egress from LNs is blocked upon inhibition or loss of S1P lyase, which increases total LN S1P almost 100 fold and down-modulates S1PR1 on T cells [30,79]. Similarly, mice lacking both SphK1 and SphK2 in lymphatic endothelial cells lack detectable S1P levels in lymph and exhibit a blockade of T cell exit from lymphoid organs [92]. After entering into the blood, naïve T cells internalize S1PR1 in response to high S1P levels in the blood, but they re-express S1PR1 during transit through secondary lymphoid organs [61]. Indeed, surface expression of S1PR1 on T cells is the primary determinant for egress kinetics [93]. In that regard, it was shown that Foxo1 controls expression of the S1PR1 transcriptional regulator KLF2 in naïve T cells and, further, that mice lacking expression of Foxo1 exhibit disrupted lymphocyte trafficking and homeostasis [72]. During lymphocyte egress from LN, whether S1PR1 promotes directional movement across the sinus endothelium or overcomes retention signals that counteract movement into the sinus is not fully understood. However, evidence is in favor of the latter model. CCL21-CCR7 signaling provides retention signals to T cells, as the cells deficient in CCR7 exit more rapidly from LNs. In contrast, CCR7-overexpressing cells were retained for longer time [82]. Furthermore, treatment of S1PR1-deficient T cells with pertussis toxin, which inactivates signaling via Gi-coupled receptors including CCR7, restored LN egress capacity to the S1PR1-deficient cells [82].

A recent study has also implicated the downregulation of KLF2 and its transcriptional target S1PR1 as a key event in the establishment of tissue resident memory T cells that develop in response to an immune stimulus and which provide long-lasting immune responsiveness in non-lymphoid tissues, including small intestines, brain, salivary gland, female reproductive tract and skin [73]. Unlike naïve CD4+ T cells, these CD8+ resident memory cells express high cell surface levels of CD69, do not express S1PR1 and do not recirculate but instead remain in nonlymphoid tissues to provide cell-mediated immunity at common sites of infection. The phenotype switch that establishes tissue resident memory T cells is initiated by cytokines including TNF, IL-33 and TGFβ, leading to downregulation of KLF2 and S1PR1 in a PI3K/AKT-dependent manner [73].

### B cell egress from BM

Unlike T lymphocyte emigration from the thymus and secondary lymphoid organs, B lymphocyte egress from BM and secondary lymphoid organs is only weakly influenced by S1P-S1PR1 signaling. is could be due to the existence of additional mechanisms such as down-regulation of CXCR4-mediated retention of B cells in BM [94,95]. In the late stages of B cell development in the BM, newly generated immature-B cells become partitioned between the BM parenchyma and sinusoids [96–98] Immature B cells are retained in the sinusoidal compartment for a short period of time before entering the blood circulation [90]. Entry of B cells into sinusoids is thought to be a critical step in egress commitment. However, the requirements for this migration event are poorly understood [99]. Pre-B cells express four of five S1PRs. S1PR1 is most abundantly expressed, whereas S1PR5 is undetectable [99]. The development of S1PR1-conditionally deficient mice [100] combined with labeling techniques [99] have provided crucial evidence that S1P signaling contributes to B cell egress. S1PR1 deficiency in B cells caused a small but significant accumulation of immature B cells in the BM parenchyma and a reduction in their frequency inside BM sinusoids and within blood [99,100]. Conversely, transgenic expression of S1PR1 in developing B cells promoted the premature egress of pro- and pre-B cells from the BM. Moreover, in vitro chemotaxis assays showed increased migration of S1PR1-transgenic Pro-B, Pre-B, and immature-B cells to S1P, indicating that S1PR1 is functional in these cells [92]. Surface expression of S1PR1 on BM B cells is negatively regulated by CD69, which inhibits the exit of these cells into the blood [48,100].

Besides S1PR1, the requirement of other S1PRs for B cell egress is not well defined. S1PR3 is differentially expressed in developing B cell subsets, being most abundant in immature IgD- and IgDlo cells. Notably, in vitro chemotactic responsiveness of immature B cells to S1P is predominantly S1PR3-mediated, suggesting the receptor is active in these cells [101]. Surprisingly, lack of S1PR3 in developing B cells led to a reduction in immature B cell frequency within BM sinuses without affecting their egress from the BM [101]. The basis for the discord between S1PR activity in vitro versus in vivo is unclear. The role of S1PR4 and S1PR2 in immature B cell egress has not been ruled out.

### Regulation of mature B cell egress from secondary lymphoid organs

Similar to T cell egress, the egress of mature B cells from secondary lymphoid organs via medullary sinuses and efferent lymphatics requires S1P-S1PR1 signaling [99,100–103]. However, evidence from in vitro chemotaxis assays suggests that migration of B cells towards S1P is independent of S1PR1 and is largely mediated by S1PR3, despite the fact that emigration of mature B cell from the BM and transit through LNs are normal in S1PR3 deficient mice [100,104]. Notably, surface expression of S1PR1 is exquisitely sensitive to low nanomolar concentrations of S1P, and the concentration of S1P used in the in vitro chemotaxis assays are very high. Nonetheless, S1PR3 is required for normal development of B cells in BM and for the positioning of transitional B cells within BM [101]. PPs play a central role in supporting B cell responses against intestinal antigens. PPs have a higher frequency of B cells [~80%] than do LNs [~30%]. B cell entry into both PPs and mucosal LNs requires α4β7 integrin and mucosal addressin cell adhesion molecule-1 [105,106]. Cyster et al., [107] discovered that B cells egress from LNs and PPs through distinct mechanisms. They have described a unique function of CXCR4 [chemokine receptor]-CXCL12 [chemokine] in B cell egress from PPs. Access of B cells to subserosal and interfollicular lymphatic vessels of PPs and their egress to lymph are promoted by CXCR4-CXCL12 mediated signaling. CXCR5-CXCL13 plays an opposing role, limiting B cell access to these sinuses and promoting B cell retention in PPs [107]. Notably, egress-promoting role of CXCR4 appears to be restricted to PPs. In addition to regulating the egress of B cells from spleen, LN and PP, trafficking of peritoneal B cells is also regulated by S1P [108].

### Regulation of marginal zone B cell shuttling

Besides regulating [109] systemic lymphocyte trafficking, S1P-S1PR1 [110] signaling is also important for the positioning of marginal zone B [MZB] lymphocytes in the spleen [55,111,112]. MZB cells are a unique B cell subset in the spleen that captures blood borne antigens and delivers them to follicular DCs, which then process and present these antigens to circulating lymphocytes [113–115]. MZB cells express S1PR1 and S1PR3, and MZB cell positioning in the MZ requires S1PR1 expression, enabling them to overcome attraction to follicular CXCL13 [55]. Movement from the MZ into the follicle is facilitated by S1PR1 desensitization mediated by a GPCR kinase, GRK2 [102]. Using intra-vital two-photon microscopy, Cyster and colleagues have visualized the movement of MZB and follicular B cells in the live mouse spleen, [111]. The major findings of this study revealed that MZB cells are highly motile and exhibit long membrane extensions. MZB cells shuttle between MZ and follicular zone, with at least one fifth of the cells exchanging between compartments per hour, a behavior that explains their ability to rapidly deliver antigens from the open blood circulation to the secluded follicles. Follicular B cells also transit from follicles to MZ. However, unlike MZB cells, follicular B cells fail to undergo integrin-mediated adhesion, become caught in fluid flow and are carried into the red pulp [111]. Localization of MZB cells to the follicular zone is disrupted by FTY720 [116,117]. MZB cells express about twofold more S1PR1 than do follicular B cells, and loss of S1PR1 causes most MZB cells to relocate from MZ to follicles. Furthermore, cells MZB cells deficient in both the receptors S1PR1 and S1PR3 exhibit uniform localization to follicles [116].

## S1P Regulation of the Trafficking of Non-lymphocytic Cells of the Immune System

### Regulation of NK cells

NK cell development occurs in the BM. After the initial stages of differentiation, NK cells undergo a maturation process and subsequently relocate throughout the body in lymphoid and non-lymphoid organs [118]. Human and mouse NK cells preferentially express S1PR5 and to a lesser extent S1PR4 [67]. In vitro studies revealed that S1PR5 acts as a non-redundant chemotactic receptor for S1P in NK cells. S1PR5 is involved in NK cell trafficking in steady-state as well as during inflammatory situations [67]. The p110α isoform of PI3K is indispensable for the chemotaxis of NK cells toward S1P [119,120]. Furthermore, S1PR5-deficient mice exhibited the accumulation of NK cells in BM and LN and a concomitant reduction in the blood, spleen, lung and inflamed liver. However, NK cells were not completely absent from these organs, suggesting the existence of other mechanisms that compensate, at least in part, for the lack of S1PR5 [67]. S1PR5 promotes NK cell exit from BM parenchyma by overcoming the CXCR4- mediated retention [14,121]. Walzer and colleagues [121] proposed that NK cell exit from the BM requires two signals, CXCR4 desensitization and activation of S1PR5. CXCR4 desensitization is not induced by S1PR5 activation, nor is the converse true. Rather, both the receptors S1PR5 and CXCR4 are sequentially desensitized by their cognate ligands to promote NK cell trafficking. NK cell exit from the LNs is not regulated by CXCR4 but is completely dependent on S1PR5 activation [121]. In a genetic screen, it was revealed that transcription of S1PR5 is regulated by T-box–containing [T-bet] transcription factor, Tbx21 [57]. Mice carrying a point mutation within Tbx21 [T-bet], exhibit a 30-fold decrease in S1PR5 transcript levels and exhibit an NK cell egress defect similar to S1PR5-deficient mice [57].

### Regulation of DC migration

DCs represent the most potent inducers of adaptive immune responses. DCs continuously acquire antigen in the periphery and migrate to draining LNs, under the influence of local environmental chemotactic factors such as CCL19/21 or S1P [59]. To trigger T cell responses, tissue-resident DCs must migrate to their draining LN. DCs emigrating into LNs exert a more mature phenotype compared with local LNs or splenic DCs [60]. S1PR1 plays a fundamental role in the migration of maturing DCs from the periphery to draining LNs. Bernhardt and colleagues [97] proposed that the contribution of different S1PRs to DC mobilization and migration is dependent on the particular DC subset under investigation. DCs undergoing maturation up-regulate S1PR1 and S1PR3 [122,123]. Both these receptors have been shown to play an indispensable role supporting the migration of DCs generated and matured in vitro [97]. However, In vivo, the migration of skin-resident DCs and intrasplenic positioning of immature DCs to the bridging channels is modulated by S1PR1 but not by S1PR3 [65,60,122]. On contrary, lamina propria DCs require S1PR1 and S1PR3 for efficient migration to the mesenteric LN [60]. In immature DCs, expression of CCR7 is low and CCL19 is unable to activate Rac1, which is required for their migration. Signaling through S1PR2 leads to Rho activation [which is known to inhibit migration] and also causes translocation of the transcriptional co-activator four-and-a-half LIM domain protein 2 [FHL2] to the nucleus, where it repress the transcription of S1pr1 to further decrease the migration of immature DCs. Maturation of DCs results in the up-regulation of CCR7 and down-regulation of S1PR2, thereby decreasing Rho activation and nuclear translocation of FHL2. In turn, this leads to increased S1P signaling via S1PR1, which promotes Rac1 activation and DC migration [48,59]. DCs also express high levels of S1PR4, and deficiency of S1PR4 can affect migration of DCs profoundly [124–126]. DCs play a pivotal role in the initiation and full manifestation of allergic diseases. Topical application of S1P and FTY720 attenuated allergic contact dermatitis reaction and experimental asthma through inhibition of DC migration to draining LN [50,98].

### S1P control of trafficking of other leukocytes

S1PR2 inhibits the migratory responses induced by S1PR1 and S1PR3 in a variety of cells [127]. S1PR2-deficient mice exhibit enhanced macrophage recruitment during thioglycollate peritonitis [127]. S1P plays a critical role in inflammation and cancer [13]. S1P lyase deficient mice exhibit elevated S1P levels in the lymphoid organs as well as in the circulatory fluid [128]. In addition to lymphocyte trafficking defect, S1P lyase deficient mice, exhibit elevated levels of pro-inflammatory cytokines and increased numbers of neutrophils in the blood. However, neutrophils from S1P lyase deficient mice show defect in chemotaxis response to S1P and failed to emigrate to inflamed tissues [128]. Deletion of the S1PR4 partially decreased the neutrophilia and inflammation in S1P lyase-deficient mice, suggesting a role of S1PR4 in neutrophil migration from blood to the tissues [128]. Similar to its role in NK cells, S1PR5 also regulates the egress of monocytes from BM. S1PR5-deficient mice lack peripheral monocytes but have a normal number of these cells in the BM [129].

HSCs are largely retained in a quiescent non-motile state in the BM niches, shifting to a migratory cycling and differentiating state to replenish the blood with mature leukocytes on demand. The balance between the major chemo-attractants CXCL12, predominantly in the BM, and S1P, mainly in the blood, dynamically regulates HSC recruitment to the circulation versus their retention in the BM. During alarm situations, stress-signals induce a decrease in CXCL12 levels in the BM, while S1P levels are rapidly and transiently increased in the circulation, thus favoring mobilization of stem cells as part of host defense and repair mechanisms [130–133].

## Summary

The complex mechanisms involved in the regulation of immune cell trafficking are of clinical importance and can be pharmacologically targeted for therapeutic benefit in the context of autoimmune disease, organ and cell transplantation and other inflammatory conditions. Many aspects of immune cell trafficking are controlled by S1P signaling through GPCRs that are differentially expressed on hematopoietic cells as well as on vascular, perivascular and stromal cells of the BM, thymus and peripheral immune organs. The most celebrated capability of S1P signaling and the one that has been successfully [134] targeted clinically involves S1PR1 signaling on mature thymic T cells. is signal enables T cells to sense and respond an S1P gradient generated by S1P catabolic enzymes that facilitates their egress from the thymus, as well as their recirculation between blood, lymph and peripheral lymphoid organs. The regulation of T cell trafficking by Camerer et al. [135] S1PR1 has been targeted successfully for the treatment of multiple sclerosis and is being investigated for potential treatment of a number of diseases and use in other clinical contexts, including but not limited to psoriasis, polymyositis, systemic lupus erythematosus, transplantation and oncology [136,137]. However S1P signaling contributes to many other aspects of adaptive and innate immune cell functions. us, the ultimate potential of targeting the S1P pathway to restrict or encourage hematopoietic cell migrations in the treatment of human disease is surely yet to be fully realized.

## Figures and Tables

**Table 1:** Important functions of S1P in the immunity.

Biological Function	Receptor Involvement	References
NFκB-TNF signaling	S1P-TRAF2 direct binding	[6]
Inflammation-induced vascular leak	S1PR1	[23]
Sepsis (PAR1-S1PR3 cross-talk in dendritic cells)	S1PR3	[80]
Differentiation of T regulatory cells	S1PR1 mediated Akt-mTOR activation	[63]
Th17 polarization	S1PR1	[37]
Thrombopoiesis	S1PR1	[135]
Mast cell activation	S1PR2	[54,87]
Anaphylaxis	S1PR2	[86]
Atherosclerosis	S1PR2	[127]

## Abbreviations

ABC: ATP-Binding Cassette
BM: Bone Marrow
DC: Dendritic Cell
DP: Double Positive
ERK: Extracellular Signal-Regulated Kinase
FHL2: Four-And-A-Half LIM Domain Protein 2
GPCR: G Protein Coupled Receptor
HDAC: Histone Deacetylase
HSC: Hematopoietic Stem Cell
JNK: C-Jun N-Terminal Kinase
KLF2: Krüppel-Like Factor 2
LN: Lymph Node
LPP: Lipid Phosphate Phosphatase
MZB: Marginal Zone B Lymphocyte
NK: Natural Killer Cell
PI3K: Phosphatidylinositol 3-Kinase
PP: Peyer’s Patches
SP: Single Positive
S1P: Sphingosine-1-Phosphate
Sphk: Sphingosine Kinase
S1PR: Sphingosine-1-Phosphate Receptor
TCR: T Cell Receptor
TPC: Thymic Progenitor Cells
TRAF2: TNF Receptor-Associated Factor 2
